# A rare case of acute rheumatic fever with three different types of atrioventricular blocks in the same patient

**DOI:** 10.14744/nci.2020.69370

**Published:** 2020-12-16

**Authors:** Kahraman Yakut, Busra Eybek, Elif Erolu, Mehmet Karacan

**Affiliations:** 1 Department of Pediatric Cardiology, Bezmialem Vakif University Faculty of Medicine, Istanbul, Turkiye; 2 Department of Pediatrics, Umraniye Training and Research Hospital, Istanbul, Turkiye; 3 Department of Pediatric Cardiology, Umraniye Training and Research Hospital, Istanbul, Turkiye; 4 Department of Pediatric Cardiology, University of Health Sciences, Umraniye Training and Research Hospital, Istanbul, Turkiye

**Keywords:** Acute rheumatic fever, atrioventricular block, arthritis

## Abstract

Acute rheumatic fever (ARF) is a systemic autoimmune disease that results from abnormal immune response to group A streptococcus pharyngitis. Although first-degree atrioventricular (AV) block is the most common rhythm problem associated with the disease, other conduction abnormalities also could be seen. We reported three different types of conduction defects (first-degree AV block, second-degree AV block, and complete AV block) in a 15-year-old case diagnosed with ARF. A 15-year-old male patient presented with palpitation. Physical examination findings were unremarkable except dysrhythmic heart sounds. Acute phase reactants were positive, and electrocardiogram showed second-degree type I AV block at hospital admission. In the 2^nd^ day of admission, right first metatarsophalangeal arthritis as well as arthralgia involved both knees and ankles developed. Echocardiography revealed moderate rheumatic mitral regurgitation. First-degree AV block with brief complete AV block episode was seen on 24 h rhythm Holter recordings. Based on clinical and laboratory findings, ARF diagnosis was made and anti-inflammatory therapy (naproxen sodium) with benzathine penicillin G was started to the patient. First-degree AV block lasted 3 weeks and other conduction disorders were not seen again first, second, and complete AV block which could be seen during ARF episode and ARF should be considered as a one of the causes of arrhythmias.

Acute rheumatic fever (ARF) is still one of the most common causes of acquired cardiac morbidity and mortality worldwide. Major and minor Jones criteria are used to diagnose the disease [1]. It is well known that ARF affects heart conduction system and frequently causes first-degree AV block which is one of Jones minor criteria. Higher degree AV block, supraventricular tachycardia, atrial/ventricular ectopic beats, bundle branch block, and accelerated nodal rhythm are other rarely reported rhythm disturbances. Although more than one conduction abnormalities were reported in the same patient with the disease, three different types of AV block in the same episode are very rare reported case to our knowledge.

## CASE REPORT

**Figure 1:**
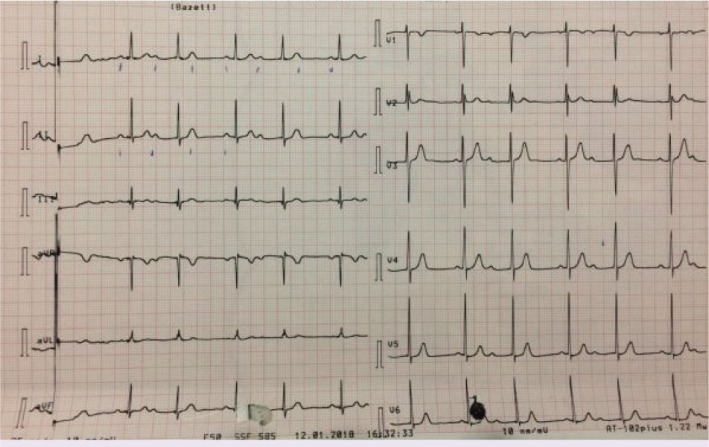
Basal ECG consistent with second-degree atrioventricular block type I.

**Figure 2:**
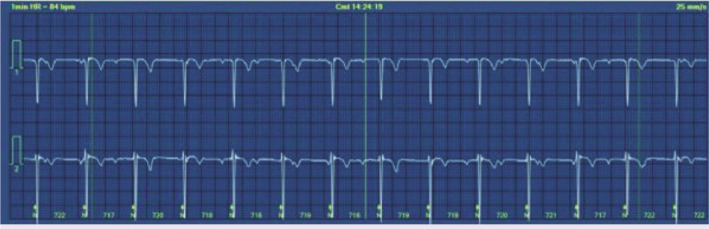
Complete atrioventricular block was revealed in 24-h rhythm Holter recordings.

**Figure 3:**
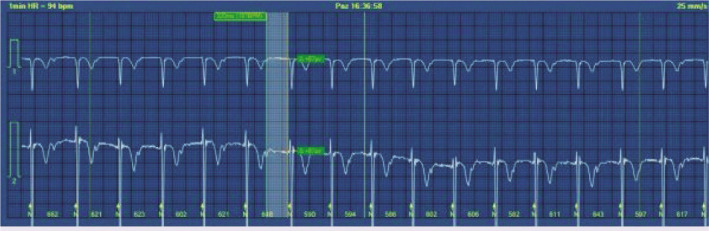
First-degree atrioventricular block pattern in was revealed in 24-h rhythm Holter recordings.

Palpitation was the chief complaint of 15-year-old male patient who visited emergency department. Two weeks ago, antibiotic was prescribed to him as he had sore throat and fever, but he was not compliant with the treatment. Physical examination findings were normal except dysrhythmia during emergency examination. Electrocardiogram showed 74 beat PER minute ventricular rate with second-degree type I AV block (Fig. 1). In medical history, tonsillectomy was performed to the patient at 6 years old due to frequent tonsillopharyngitis, and patient’s mother had rheumatic heart disease. In the laboratory studies, C-reactive protein was 13 mg/dl (Normal range: <5 mg/dl), anti-streptolysine O was 599 IU/ml (Normal range: <250 IU/ml) and erythrocyte sedimentation rate was 77 mm/h. Echocardiography revealed moderate rheumatic mitral valve regurgitation. Cardiothoracic ratio was 47% on the chest X-ray. Arthralgia on both knees and ankles as well as right first metatarsophalangeal arthritis developed on the 3^rd^ day of admission. Fulfilled two major, one minor criteria in the presence of supporting evidence of group A streptococcus infection, the case was diagnosed with ARF. Anti-inflammatory therapy with naproxen sodium (20 mg/kg/day, BID) and antibiotic therapy with benzathine penicillin G were started. Brief episode of complete AV block was seen on 24 h rhythm Holter (Fig. 2) and the rest of the record demonstrated marked first-degree AV block [(Fig. 3), PR interval: 320 msn]. Prompt resolution of joints manifestations was observed in the 2^nd^ day of anti-inflammatory therapy. The PR interval was prolonged during the acute phase of the disease, but PR interval shortened (135 ms) after normalization of acute phase reactants.

One week after discontinuation of anti-inflammatory treatment, PR interval prolonged to 220 ms again meanwhile acute phase reactants were elevated. These findings were explained with ARF relapse. Anti-inflammatory therapy was resumed and administered for 3 weeks. PR interval returned to normal once acute phase reactants were normal. This case has received penicillin prophylaxis since he was diagnosed with ARF and his outpatient visits have been made regularly for 6 months. During follow-up, dysrhythmias was not seen again neither on ECG nor on 24-h rhythm Holter recordings. Informed consent was obtained from patient and family for this study.

## DISCUSSION

ARF remains the most common cause of acquired heart disease in some countries in the world. The most common conduction abnormality associated with the disease is first-degree AV block which is also one of minor criteria [2–4]. The incidence of first-degree AV block during ARF episode has been reported between 34.2% and 72.3% [5, 6]. In addition to first-degree AV block, advanced degree AV blocks, junctional rhythm, premature atrial contractions, ventricular extrasystoles, and ventricular/supraventricular tachycardias were observed in ARF patients [2–9]. Zalstein et al. [7] found first-degree AV block in 72.3% of patients, Mobitz type I AV block in 1.5% and complete AV block in 4.6% of patients in their study. Agnew et al. [8] demonstrated various type of transient AV conduction abnormalities in 8.5% of patients with the rate of second- and third-degree AV block 2.5%. Furthermore, nodal rhythm was seen in 6% of patients in the same study. It was rarely reported cases who experienced both Mobitz type II and third-degree AV block in the same ARF episode [7, 10]. Karacan et al. [2] showed that analyzing 24-h rhythm Holter recordings demonstrated more ARF patients had rhythm disturbances than had been previously recognized. We identified third-degree AV block in our patient by evaluating rhythm Holter recordings.

Although the mechanism of AV block in ARF has not been fully understood, it has been speculated that an increase in vagal tone and immunologic effect on AV node could be two causes. Conduction abnormalities associated with ARF are usually self-limited and improve in several weeks after initiation of non-steroidal anti-inflammatory therapy [8, 10, 11]. Some studies have reported a good response to corticosteroids [12, 13]. This shows that corticosteroids may be a good option in the treatment of advanced degree AV block due to ARF. On the other hand, permanent pacemaker implanted cases with AV block that caused syncope and hemodynamic instability were reported both in adult and pediatric literature [14, 15]. As literature showed rhythm problems of our cases resolved with the anti-inflammatory treatment.

We presented an uncommon case diagnosed with ARF and three types of AV block. The lesson we learnt from this experience is that ARF should come to mind when one encounter with particularly first-degree AV block but also other type of AV blocks. 24 h rhythm Holter monitorization is a useful tool to reveal rhythm abnormalities in ARF patients.
